# Structure elucidation and docking analysis of 5M mutant of T1 lipase *Geobacillus zalihae*

**DOI:** 10.1371/journal.pone.0251751

**Published:** 2021-06-01

**Authors:** Siti Nor Hasmah Ishak, Nor Hafizah Ahmad Kamarudin, Mohd Shukuri Mohamad Ali, Adam Thean Chor Leow, Fairolniza Mohd Shariff, Raja Noor Zaliha Raja Abd Rahman

**Affiliations:** 1 Enzyme and Microbial Technology Research Centre, Faculty of Biotechnology and Biomolecular Sciences, Universiti Putra Malaysia, Serdang, Selangor, Malaysia; 2 Department of Microbiology, Faculty of Biotechnology and Biomolecular Sciences, Universiti Putra Malaysia, Serdang, Selangor, Malaysia; 3 Centre of Foundation Studies for Agricultural Science, Universiti Putra Malaysia, Serdang, Selangor, Malaysia; 4 Department of Biochemistry, Faculty of Biotechnology and Biomolecular Sciences, Universiti Putra Malaysia, Serdang, Selangor, Malaysia; 5 Department of Cell and Molecular Biology, Faculty of Biotechnology and Biomolecular Sciences, Universiti Putra Malaysia, Serdang, Selangor, Malaysia; 6 Institute of Bioscience, Universiti Putra Malaysia, Serdang, Selangor, Malaysia; 7 Laboratory of Halal Science Research, Halal Products Research Institute, Universiti Putra Malaysia, Serdang, Selangor, Malaysia; Government College University Faisalabad, PAKISTAN

## Abstract

5M mutant lipase was derived through cumulative mutagenesis of amino acid residues (D43E/T118N/E226D/E250L/N304E) of T1 lipase from *Geobacillus zalihae*. A previous study revealed that cumulative mutations in 5M mutant lipase resulted in decreased thermostability compared to wild-type T1 lipase. Multiple amino acids substitution might cause structural destabilization due to negative cooperation. Hence, the three-dimensional structure of 5M mutant lipase was elucidated to determine the evolution in structural elements caused by amino acids substitution. A suitable crystal for X-ray diffraction was obtained from an optimized formulation containing 0.5 M sodium cacodylate trihydrate, 0.4 M sodium citrate tribasic pH 6.4 and 0.2 M sodium chloride with 2.5 mg/mL protein concentration. The three-dimensional structure of 5M mutant lipase was solved at 2.64 Å with two molecules per asymmetric unit. The detailed analysis of the structure revealed that there was a decrease in the number of molecular interactions, including hydrogen bonds and ion interactions, which are important in maintaining the stability of lipase. This study facilitates understanding of and highlights the importance of hydrogen bonds and ion interactions towards protein stability. Substrate specificity and docking analysis on the open structure of 5M mutant lipase revealed changes in substrate preference. The molecular dynamics simulation of 5M-substrates complexes validated the substrate preference of 5M lipase towards long-chain *p*-nitrophenyl–esters.

## Introduction

Lipase (triacylglycerol acylhydrolase E.C.3.1.1.3) is a class of enzymes belonging to the serine hydrolases and is widely known as important biocatalysts. Lipase has been broadly used to catalyze various reactions such as hydrolysis, esterification, interesterification, alcoholysis, aminolysis, acidolysis, and lipolysis [1–7]. The enzyme is produced by various organisms including animals, plants, bacteria, fungi, and yeast [8–10]. As a leading biocatalyst, a lipase needs to offer stability and flexibility while effectively catalyzing broad reactions in organic solvents, high temperatures, and salinity. Organic solvent and temperature tolerant lipase are important in the application of meat degradation, fatty acid ester synthesis, biodiesel processes as well as food and detergent industries [11–14]. These enzymes allow catalytic reactions to be conducted at higher temperatures and withstand denaturation, which is the main cause of enzyme deactivation [15].

The substitution of amino acid in the primary sequence may significantly improve the stability of the enzyme. This amino acid substitution was found to change the conformation of the protein involved in the additional interaction between amino acids of protein. For example, the formation of the disulfide bridge in alkaline α-amylase mutants produced from *Alkalimonas amylolytica* has been shown to improve enzyme stability at a high temperature [16]. In addition, hydrogen bonds and ionic interactions formed between different side-chain groups contribute to protein stability. Hydrogen bonds were observed to connect β3, β5, loop, and 310-helix closely in structure of 6B mutant of *Bacillus subtilis* lipase (Lip A), hence stabilize its structure [17]. Increasing in the number of ionic interactions in mutant structure of L2 lipase (S385E) of *Bacillus* sp. helped stabilize the structure of the lipase [18]. The ability of a protein to preserve the secondary structure is important for functional sustainability. Because proteins denature rapidly under extreme conditions, it is important to restore the tertiary structure through its shape and folding patterns.

The 5M mutant lipase derived from genetically modified of T1 lipase *Geobacillus zalihae* consists of 388 amino acids with a molecular weight of 43 kDa. This lipase has five mutations (D43E, T118N, E226D, E250L, and N304E) which have been genetically modified based on the comparison of earth- and space-grown T1 lipase structures [19, 20]. This mutant lipase showed similar characteristics in temperature and pH optimum as wild-type T1 lipase, which optimally active at temperature 70°C and pH 9. The mutations ensuing in 5M lipase resulted in increased catalytic efficiency in DMSO, methanol, and n-hexane compared to the wild-type T1 lipase. However, the comparison of thermostability with its parent lipase indicates that 5M lipase has a low tolerance against high temperature based on the half-life study, and melting point analysis. Analysis of the model structure of 5M lipase suggests that the number of molecular interactions such as hydrogen bonds and ion interactions have decreased due to the mutations [20]. Thus, subsequent study has been conducted to solve the three-dimensional structure of 5M lipase and revealed the factor contributes to the decrease in lipase stability using the crystal structure of 5M lipase. In addition, the selectivity and substrate specificity of 5M mutant lipase will also be discovered.

## Materials and methods

### Protein preparation and purification

The cumulative mutation of five amino acids (D43E/T118N/E226D/E250L/N304E), namely as 5M mutant lipase was constructed via gene synthesis (Bio Basic Inc., Canada). The synthesized oligonucleotide was subsequently cloned into modified plasmid pGEX/4T1 with His-tag [21] and was expressed in *Escherichia coli* BL21(DE3)pLys*S*. Mutant 5M lipase was cultured in 1 L Luria Bertani (LB) broth at 37°C overnight at 150 rpm. The culture was induced with 0.025 mM isopropyl-β-D-thiogalactoside (IPTG) once the OD_600_ reached 0.6–0.7 prior the cultivation. After 12 hours, the fermentation broth was harvested at 10,000 x*g* for 30 minutes. The cell pellet was re-suspended in 100 mL binding buffer (20 mM sodium phosphate, 0.5 M NaCl, and 0.5 mM imidazole, pH 7.4) supplemented with 5 mM dithiothreitol (DTT) prior to sonication. The sonicated protein was centrifuged at 10,000 x*g*, 4°C for 30 minutes, and the crude extract of protein solution was loaded into a 16XK column containing a tightly packed Ni-Sepharose fast flow resin (GE Healthcare, Sweden) via 1 mL/min flow rate. The bound protein was eluted with 20 mM sodium phosphate, 0.5 M NaCl, and with a gradient of 0–0.5-M imidazole, pH 7.4.

Ion exchange chromatography was applied as the second step purification to polish the purified protein prior to crystallization. The pool fraction of eluted protein was dialyzed overnight for buffer exchange and His-tag removal by thrombin (1 U/mg of fusion protein). The dialyzed protein was loaded into 16XK column containing Q-Sepharose fast flow resin (GE Healthcare, Sweden). The resin was equilibrated by a binding buffer (25 mM Tris-HCl, pH 9.0) before protein was loaded. The washing step was performed until there was no protein detected, and the bound lipase was eluted via gradient with elution buffer 25 mM Tris-HCl pH 9.0 supplemented with 0.5 M NaCl.

### Crystal screening and optimization

Crystal Screen kits (Hampton Research, USA) and JCSG-*plus* Box 1 and Box 2 (Molecular Dimensions, UK) were used for crystal screening of 5M mutant lipase using the sitting drop vapor diffusion method. The concentrations of protein were set to 5 mg/mL in 25 mM Tris-HCl pH 9.0 supplemented with 0.15 M NaCl. The drop contains 2 μL of solution with ratio 1:1 protein to the reservoir solution and were equilibrated against 100 μL of reservoir solution at 15°C. Based on the screening results, formulation 2 (1.0 M sodium citrate tribasic dehydrate, 0.1 M sodium cacodylate, pH 6.5) from JCSG-*plus* box 2 was selected for subsequent optimization. The purified protein of 5M lipase at a concentration of 5 mg/mL was tested on various concentrations of sodium cacodylate trihydrate, and sodium citrate tribasic trihydrate on crystal growth, with concentrations from 0.1 to 0.5 M. Further, the quality of the crystal was improved by optimizing the protein and salt concentrations. Protein at various concentrations (1.0, 1.5, 2.0, 2.5, 3.0, 3.5, 4.0, 4.5, and 5.0 mg/mL) was used for crystal growth optimization using the formulation of 0.5 M sodium cacodylate trihydrate, and 0.4 M sodium citrate tribasic trihydrate. The optimized protein concentration (2.5 mg/mL) was applied in subsequent optimization using sodium chloride (NaCl) at concentrations of 0.025, 0.05, 0.1, 0.2, and 0.25 M. The drop contains 3 μL protein reservoir solution exhibiting ratios 1:1 and was equilibrated with 1 mL of reservoir solution. The mixture was incubated at a temperature of 15°C and the growth of the crystal was observed.

### X-ray diffraction, data collection and data processing

The single formation of crystal was selected for X-ray diffraction and data collection. The 5M mutant lipase crystal was mounted using 0.3 mm cryoloops (Hampton Research, USA) and was cooled to 100 K in a nitrogen stream. The data were collected using an in-house X-ray diffractometer (Rigaku, Japan) and were indexed, integrated, and scaled. In order to obtain a good model fitting both geometry requirements and the X-ray diffraction data, a refinement was performed using a rigid body (REFMAC5) from the CCP4 package [22]. CCP4 Program Suite and COOT (Crystallographic Object-Oriented Toolkit) [23] softwares were applied in data processing for structure refinement, validation, and visualization of the electron density map. The model building step involved manual fitting of the molecule parts into the electron density map. The refinement process involved alternating rounds of automated optimization and manual corrections of the model to improve its agreement with the electron density maps. The refinement step involved the adjustment of the position and temperature factor of all atoms in the model, besides locating water molecules and ions. The R_free_ and R_factor_ were observed for agreement between observed and predicted structure factors. 5M mutant crystal structure was validated using the Ramachandran plot, ERRAT, and Verify_3D tools [24, 25] via SAVES v5.0 online software (https://servicesn.mbi.ucla.edu/SAVES/). The structure factors and atomic coordinates have been submitted for inclusion in the Protein Data Bank (www.rcsb.org/) with the accession code 7BUK.

### Overall structure and secondary structure analysis of 5M mutant crystal structure

YASARA software was applied for visual analysis and structure inspection. The analysis of secondary structure of 5M mutant was performed using PDBsum [26], a web-based database of summaries and analyses of PDB structures. The PDB file of 5M was uploaded into the database and secondary structure elements such as β-sheets, β-hairpins, helices, β-turns, and γ-turns were analyzed. The crystal structure of 5M was superimposed with its native crystal structure T1 lipase (PDB ID: 2DSN). The chemical interactions including, the intramolecular and intermolecular interactions, such as hydrophobic interactions, hydrogen bonds, ionic interactions, and cation-π interactions were determined and compared with the molecular interactions in earth-grown T1 lipase structure.

### Substrate specificity and hydrolysis in various natural oils

Substrate specificity of 5M mutant lipase was determined by using the different chain length of *p*-nitrophenyl–esters (*p*NP) includes *p*NP-acetate (C2), *p*NP-butyrate (C4), *p*NP-decanoate (C10), *p*NP-dodecanoate (C12), *p*NP-myristate (C14), and *p*NP-palmitate (C16). The reaction mixture containing 188 μl of 50 mM Tris-HCl pH 9.0, 10 μl of the substrate (0.05 mM), and 2 μL of enzyme solution was assayed at 70°C for 5 minutes prior to the termination by using ethanol. The lipase activity was determined by measuring the absorbance of liberated *p*-nitrophenol at 405 nm. One unit of activity was defined as the rate of enzyme needed to release 1 μmol of *p*-nitrophenol per minute.

The hydrolytic activity of 5M lipase toward various natural oils were measured by colorimetric assay described previously [22]. The substrate preference of lipase was tested using natural oils, such as coconut oil (C12:0), olive oil (C18:1), rice bran oil (C18:1), soy oil (C18:1), corn oil (C18:2), sunflower oil (C18:2), and canola oil (C18:1) with the emulsion of each oil and buffer ratio 1:1. The mixture of a solution containing 10 μL of the enzyme, 20 μL of 20 mM calcium chloride (CaCl_2_), 2.5 mL of the substrate, and 990 μL of buffer was assayed at 70°C for 30 minutes with 200 rpm agitation. The lipase activity was calculated by measuring the absorbance at 715 nm. One unit (U) is the rate of enzyme catalyzes the reaction of 1 μmol substrate per minute. The activity of lipase in olive oil was set as 100%.

### Molecular docking

The open structure of 5M mutant lipase was modeled using *Geobacillus thermocatenulatus* lipase (PDB ID: 2W22) as a template (96% identity). The ligands (pNP-C2 to–C16) were retrieved from the PubChem database (https://pubchem.ncbi.nlm.nih.gov/). The C2 to C16 p-nitrophenyl substrates was docked into 5M mutant structure to study the ability of 5M mutants in the catalysis of substrates with different chain lengths. Protein–ligand docking of 5M mutant with different lengths of substrates was investigated by YASARA (Yet Another Scientific Artificial Reality Application) package, using autodock VINA and AMBER03 force field [27]. Water molecules were removed during protein preparation. The simulation cell was generated around the catalytic site of 5M. Energy minimization was performed on the ligand structure using simulated annealing prior docking analysis. The analysis was performed for 25 runs. The docking results were analyzed using the LigPlot+ [28].

### Molecular dynamics simulation

The 5M-C10 and 5M-C14 protein-ligand complexes with the highest binding energy and the 5M-C2 protein-ligand complexes with the lowest binding energy were selected for molecular dynamics simulation along with the free 5M lipase crystal structure (7BUK). Molecular dynamics simulations of the protein-ligand complexes were performed using YASARA software along with AMBER14 force field. The protein structures were solvated with water molecules in a periodic box, maintaining the density of 0.997 g/L. The systems were minimized using the steepest descent algorithm without electrostatic interaction to remove the conformational stress followed by equilibration. The systems were simulated under physiological conditions (pH 7.0, 0.9% NaCl) and the optimum temperature of 5M lipase (343.15 K). MD simulations were performed for 50 ns long using the md_run.mcr script in YASARA and MD trajectories were saved every 25 ps for further analysis. After the completion of simulations, the RMSD (root mean square deviation), RMSF (root mean square fluctuation), radius of gyration (Rg) and binding energy of the trajectories were analyzed.

## Results and discussions

### Purification and crystallization of 5M lipase

**Fig 1:**
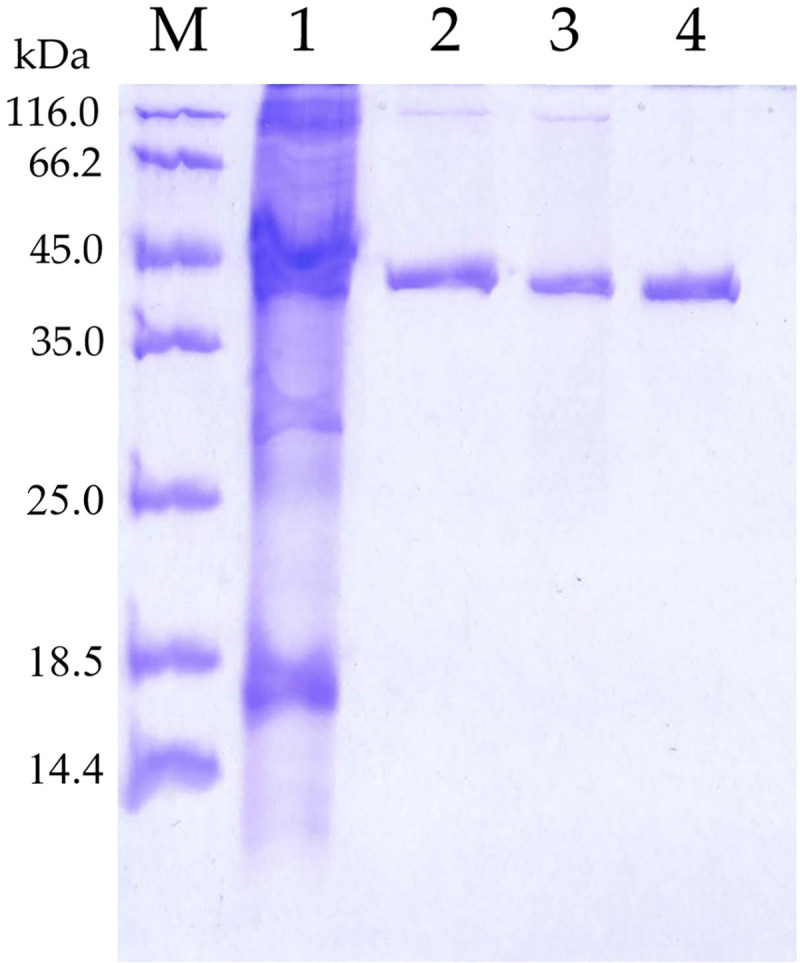
SDS-page analysis of 5M mutant lipase. M, protein marker. Lane 1, crude enzyme. Lane 2, pool fraction of affinity chromatography. Lane 3, tag-cleaved 5M mutant lipase. Lane 4, purified 5M mutant lipase after anion exchange chromatography. Approximately 10 μg of protein sample was loaded into the well.

The 5M mutant is a hydrolase enzyme derived from genetically modified T1 lipase of *Geobacillus zalihae* in the aim of introducing extra hydrogen bonds and ion interactions in its structure. The molecular mass and pI value of 5M without His-tag is 43 kDa and 5.9. The pI of 5M mutant lipase was comparatively lower than its wild-type HT1 lipase, which has a pI value of 6.1. Previously, biochemical characterization was conducted across parameters, such as temperature, pH, metal ions, and organic solvents. The stability of 5M mutant lipase in methanol was enhanced compared to the wild-type HT1 although, 5M mutant decreased in stability at high temperatures [20]. The two-step purification procedure showed a single band of protein with high homogeneity (Fig 1).

**Fig 2:**
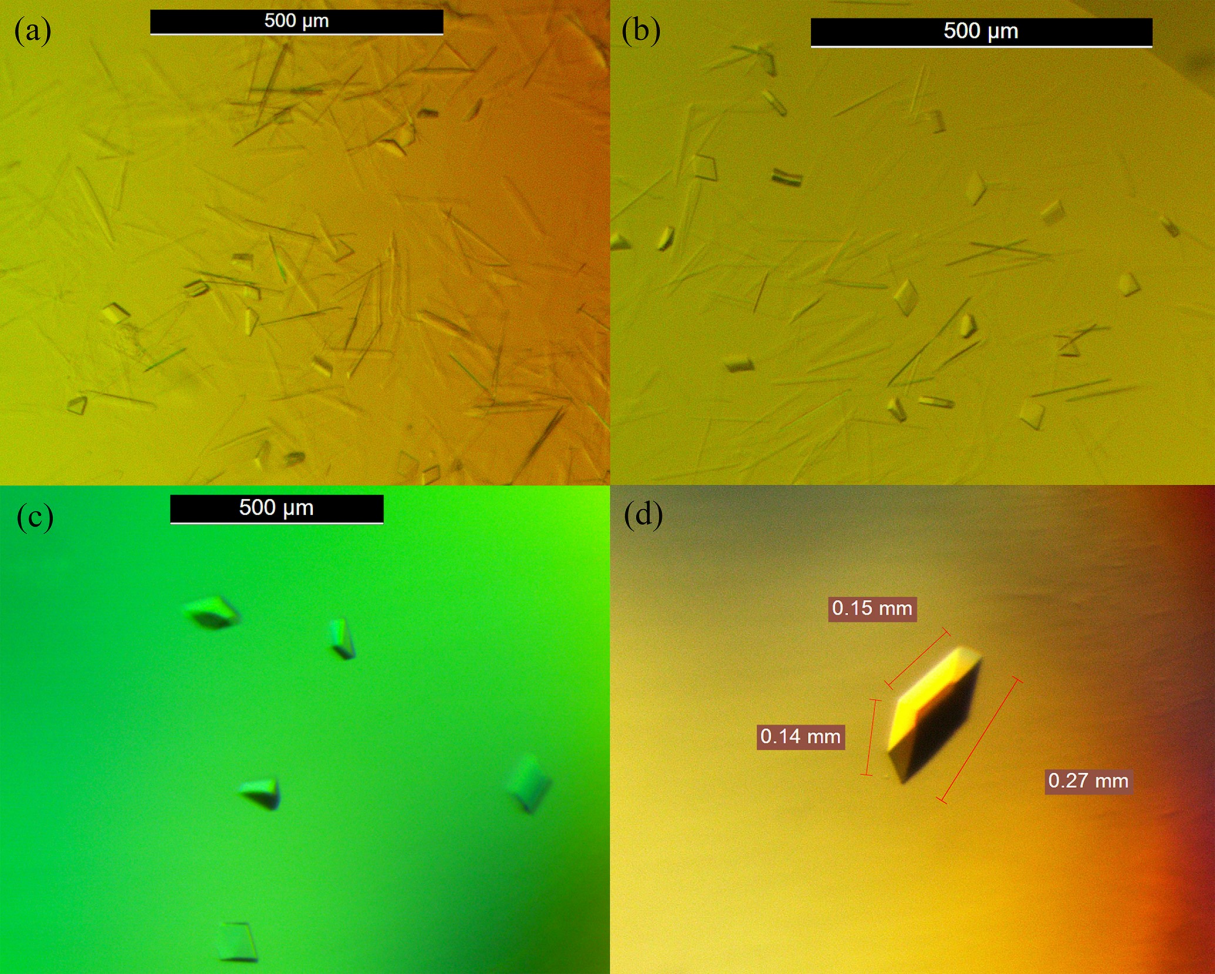
5M mutant crystals. (a) Crystal of 5M mutant observed after one week of incubation during crystal screening with protein concentration of 5 mg/mL. (b) Crystal observed in the optimized formulation of 0.5 M sodium cacodylate pH 6.5, 0.4 M sodium citrate tribasic dehydrate with a protein concentration of 5 mg/mL. (c) The morphology of the crystal improved after the optimization of protein concentration. The best concentration was at 2.5 mg/mL. (d) Good morphology and shape of the crystal obtained after the addition of salt. Crystal obtained at a concentration of 0.2 M sodium chloride.

At the protein concentration of 5 mg/mL, the formation of protein crystals was observed after 2 days of incubation at 15°C. The crystal hits were able to develop in 24 formulations from Crystal Screen Kits (Hampton Research, CA, USA) with another 40 types of formulations from JCSG-plus Kits (Molecular Dimensions, OH, USA). Following the observation of crystal formations using formulation 1 from JCSG-*plus* (1.0 M Sodium citrate tribasic dehydrate, 0.1 M Sodium cacodylate trihydrate pH 6.5), the formation of a three-dimensional crystal was found to be most convincing and was later applied in crystal optimization (Fig 2A).

The formulation using a low concentration of sodium cacodylate trihydrate and sodium citrate tribasic caused an increased formation of needle and plate-shaped crystals. However, when the concentration of both buffers was increased, the crystal formation showed a combination of needle and rhombohedron crystal shapes (Fig 2B).

Attempts to improve the quality of the 5M mutant lipase crystal interface using a higher concentration of protein (10 mg/mL) were unsuccessful. A high concentration of protein led to the formation of many microcrystals due to the high rate of nucleation. The best protein concentration for the 5M mutant lipase crystal formation was obtained at 2.5 mg/mL in the optimized formulation containing 0.4 M sodium citrate tribasic and 0.5 M sodium cacodylate trihydrate pH 6.5 by sitting drop method after a day of incubation (Fig 2C). Protein concentrations higher than 3.0 mg/mL produced many needle plate-shaped crystals. Lower protein concentrations such as 1.0 and 1.5 mg/mL exhibit a clear drop and no crystals. By using 2.0 mg/mL of protein, few hits with only rhombohedron-shaped crystals were produced after 7 days of the incubation period. The results indicated that lower protein concentrations would lead to a longer time to develop a crystal compared to high concentrations, whereby the protein at the concentration of 5 mg/mL produced crystal as fast as one day of incubation. Macromolecule crystallization is produced mostly at its optimum concentration from 8 to 20 mg/mL. Ideally, a small and medium sized protein required concentrations of 30 mg/mL or more, whereas a bigger sized protein or macromolecules required lower concentrations of protein that were around 3 to 5 mg/mL to develop a crystal [29]. Accordingly, for 5M lipase, any concentrations of protein above 4 mg/mL resulted in rapid nucleation that led to the formation of microcrystals. The growth of disordered crystals was attributed to the uncontrolled and rapid nucleation of the crystal having overload protein concentration [29]. Similarly, Mazlan et al. [30] verified that at greater than 10 mg/mL concentrations of protein, the quality of the crystal from GDSL esterase (EstJ15) of *Photobacterium* sp. was found not to be significantly improved.

The addition of an ionic strength agent precipitation, such as sodium chloride (NaCl) into the base formulation, which was free from precipitant or salt could improve the quality of the crystal. The results showed that by adding sodium chloride into the said formulation, the shape and size of protein crystals could be improved, as shown in Fig 2D. Moreover, a high concentration of NaCl or salt (0.2 and 0.25 M) could produce a three-dimensional crystal having a good shape with a cleaner cut. The salt concentration is important because it affects the solubility of the protein solution. Given that the salt concentration is too low, the concentration in the drop may not reach the marginal concentration level required for crystallization. However, at high salt concentration, the protein may experience declining in protein solubility. An ideal salt concentration will slowly eliminate water components from the drop solution as well as to increase the concentration of protein in a slow way [31]. According to McPherson and Cudney [29], it is common that a salt concentration above 0.2 M would increase the solubility of protein but slows down the growth of the crystal to keep the crystallization process under control. With a high protein concentration, the mutant 5M lipase faced uncontrolled nucleation, where the crystals were rapidly produced after one day of inoculation. This condition leads to the production of many microcrystals, which are not suitable for X-ray diffraction. By reducing the protein concentration to 2.5 mg/mL and adding an ideal concentration of sodium chloride, the growth of crystals can be significantly improved. Results from the crystallization process demonstrate that the addition of sodium chloride at 0.2 M and 0.25 M encourages a decline of the nucleation rate and improves the overall size of the crystal along with the process slowly. According to Leow et al. [32], the concentration of protein above 2.5 mg/mL supplemented with precipitant lower than 1 M NaCl stimulates a larger crystal formation.

### Overall structure of 5M lipase

**Fig 3:**
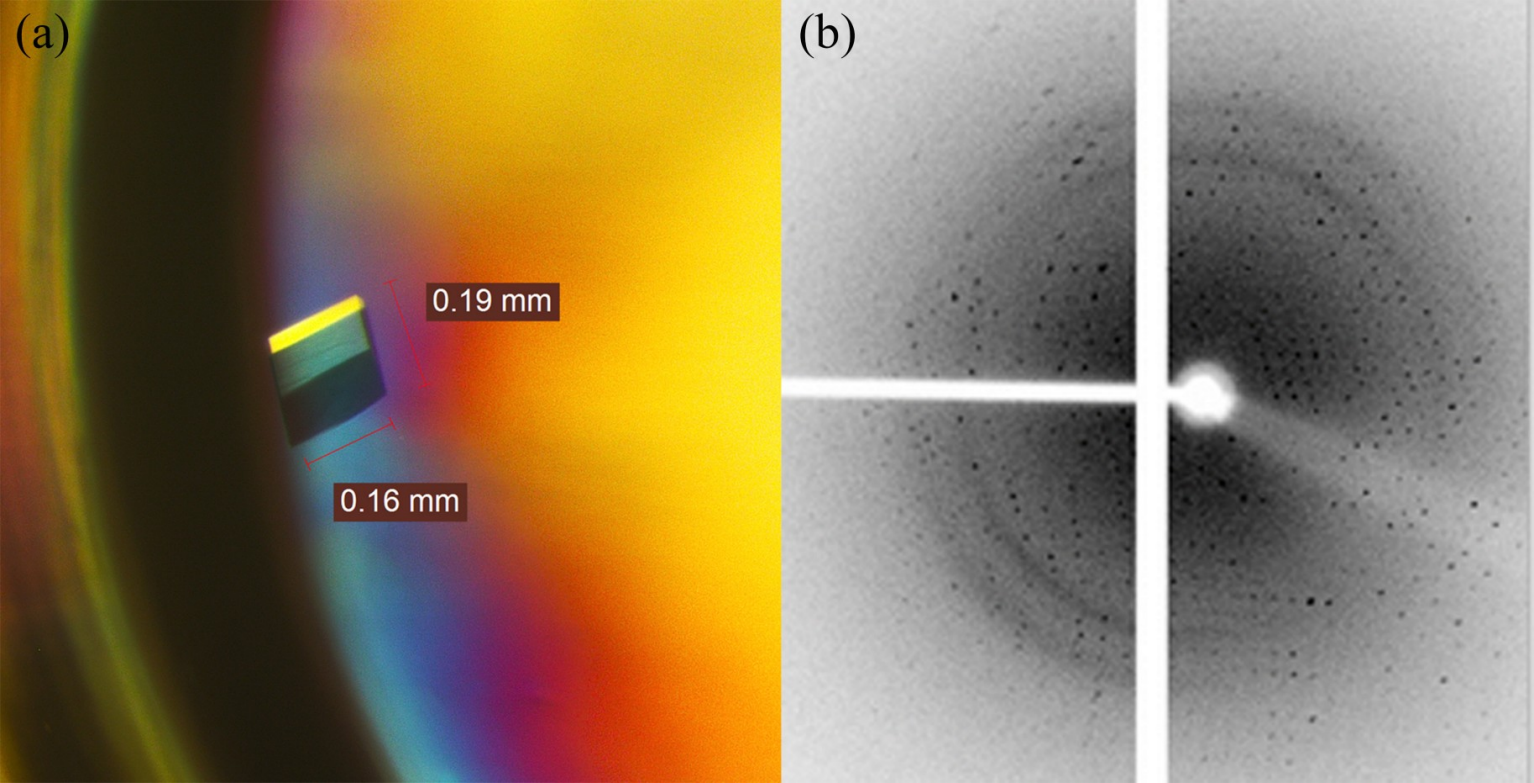
5M mutant diffraction analysis. (a) 5M mutant crystal structure formed in the formulation of 0.5 M sodium cacodylate trihydrate and 0.4 M sodium citrate tribasic pH 6.5 supplemented with 0.2 M NaCl. (b) Diffraction image of 5M mutant lipase crystal.

**Fig 4:**
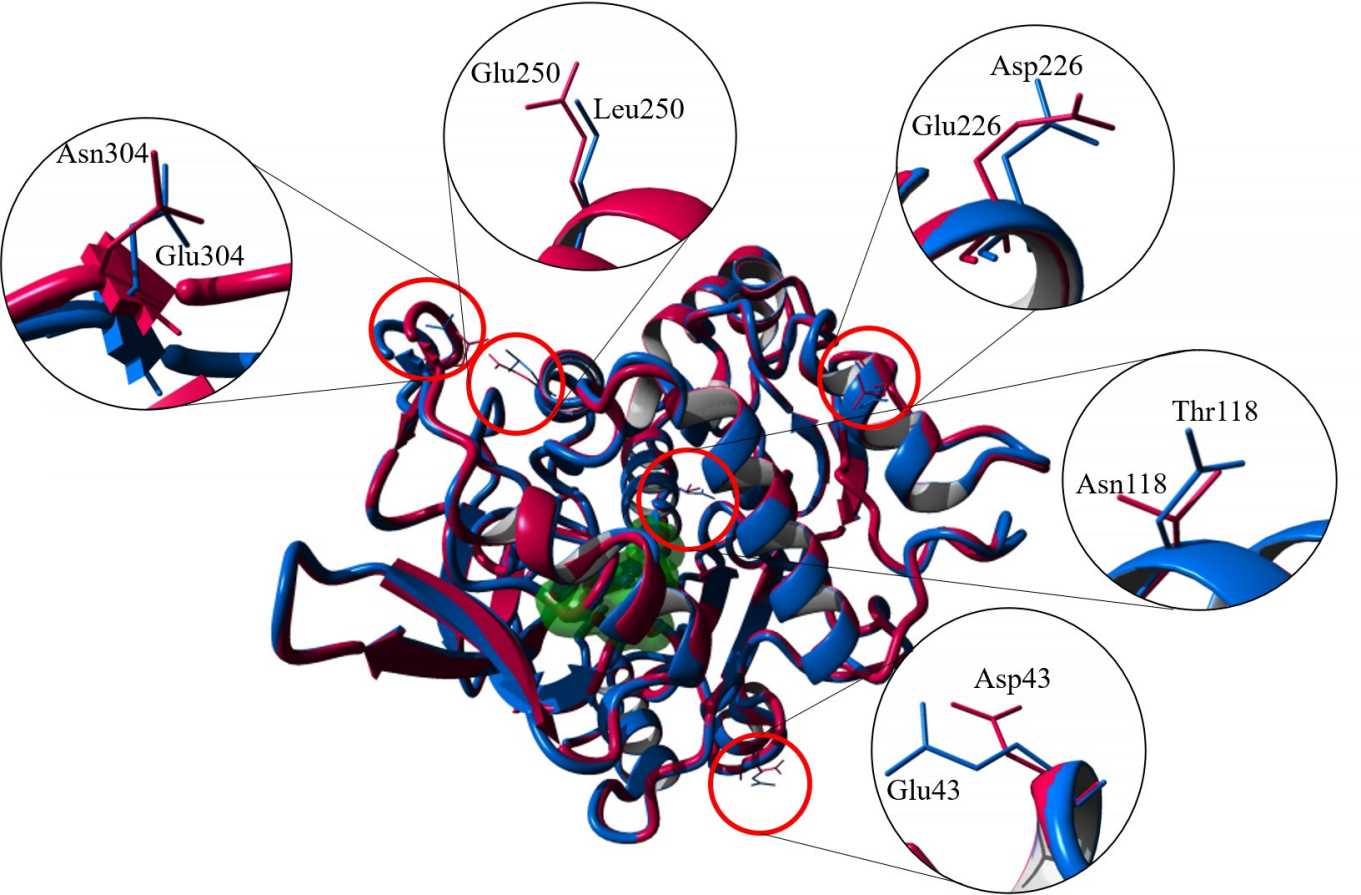
Superimposed of lipase crystal structure 5M mutant lipase (7BUK) and its native structure, T1 lipase (2DSN). The location of the mutations was highlighted in the red circle.

The data set was collected from a good morphology single protein crystal with approximate dimensions of 0.16 x 0.19 mm obtained from the optimized conditions (Fig 3). The statistics of data collection and refinement of 5M mutant lipase was summarized in Table 1. The crystal structure of 5M lipase was solved at a resolution of 2.64 Å with 90.9% completeness. After refinement, the R_factor_ and R_free_ values of 5M mutant crystal structure reached 0.184 and 0.262, respectively. The crystal contains two molecules per asymmetric unit, similar to the reported wild-type of T1 lipase crystal structure (2DSN) [33]. The superimposed of 5M mutant crystal structure and 2DSN shows RMSD of 0.728 Å (Fig 4). The 5M crystal fitted the monoclinic system space group C2, signifying unit cell parameters of *a* = 117.59 Å, *b* = 81.27 Å, *c* = 99.52 Å. Other lipases, such as a thermostable lipase from *Bacillus stearothermophilus* P1 [34], reportedly have the same space group with the present 5M lipase, as the space group was found to be monoclinic. The 3D crystal structure of 5M mutant lipase was validated using the Ramachandran plot, Verify-3D, ERRAT, and Procheck via SAVES server online tools. In the Ramachandran plot, a good quality model is obtained if the model has more than 90% of its residues in the most favored region [25]. For the 5M lipase mutant, the generated Ramachandran plot indicated that 89.4% of the residues lie in the most favored region and another 9.8% residue situated in the additionally allowed region. Meanwhile, about 0.3% of residues were found to be in the disallowed region. Catalytic serine residues (Ser113) on both chain A and chain B lie within the generously allowed region showing a typical conformation of the “nucleophilic elbow”. This residue was positioned in a firmly constrained beta-turn type structure located between a β-strand and an α-helix similarly displayed by a crystal structure of lipase from *Bacillus stearothermophilus* P1 [34]. The Verify-3D analysis showed that about 99.74% of the residues had an average 3D-1D score of more than 0.2. The accuracy of a structure model can be verified by measuring the compatibility of a protein model and its sequence by using a 3D profile [35]. The high score obtained from the Verify-3D analysis indicates a properly developed protein structure. The ERRAT analysis indicated that 5M mutant crystal structure can be accepted as a good model structure, as the overall quality factors reached 96.6% and 94.2% for chains A and B, respectively.

**Table 1:** Data statistic of X-ray diffraction analysis.

PDB ID	7BUK
Data collection	
Space Group	C 1 2 1
a, b, c (Å)	117.59, 81.27, 99.52
*α*, *β*, *γ* (°)	90.0, 97.3, 90.0
Wavelength (Å)	1.54178
Resolution range (Å)	35.00–2.64 (2.70–2.65)
No. of observed reflections	53286
No. of unique reflections	24822
Linear R-Merge	0.056(0.129)
Average *I*/σ	14.0(5.2)
Completeness (%)	90.9(81.4)
Average Redundancy	2.1 (1.7)
Refinement	
No. of protein atoms	774
No. of water molecules	183
No. of ligands	4
R.m.s. deviation, bond lengths (Å)	0.0085
R.m.s. deviation, bond angles (°)	1.587
All atoms average B factor (Å ^2^)	17.0
R_factor_	0.184
R_free_	0.262
Ramachandran plot (%)	
Most favored regions	89.4
Additional allowed regions	9.8
Generously allowed regions	0.5
Verify-3D (%)	99.74
ERRAT (%)	
Chain A	96.6
Chain B	94.2

Values in parentheses are for the highest resolution shell.

**Fig 5:**
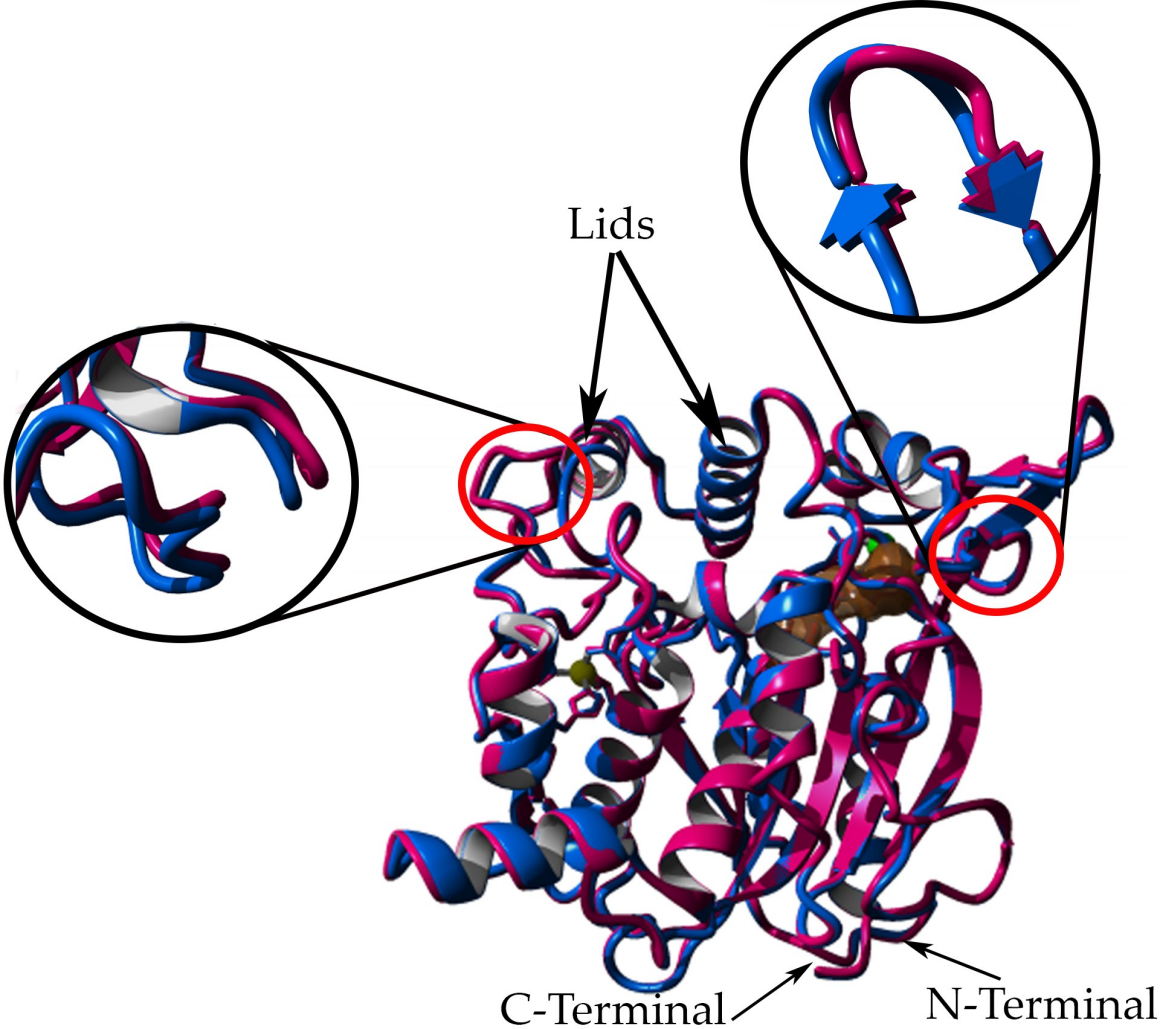
Structural alignment of 5M (7BUK) chains A and B. Chain A colored in magenta, chain B colored in blue, yellow ball represents a zinc ion, the green ball represents calcium ion, brown surface residues represent the catalytic triad of lipase. The image was generated via YASARA [27].

**Fig 6:**
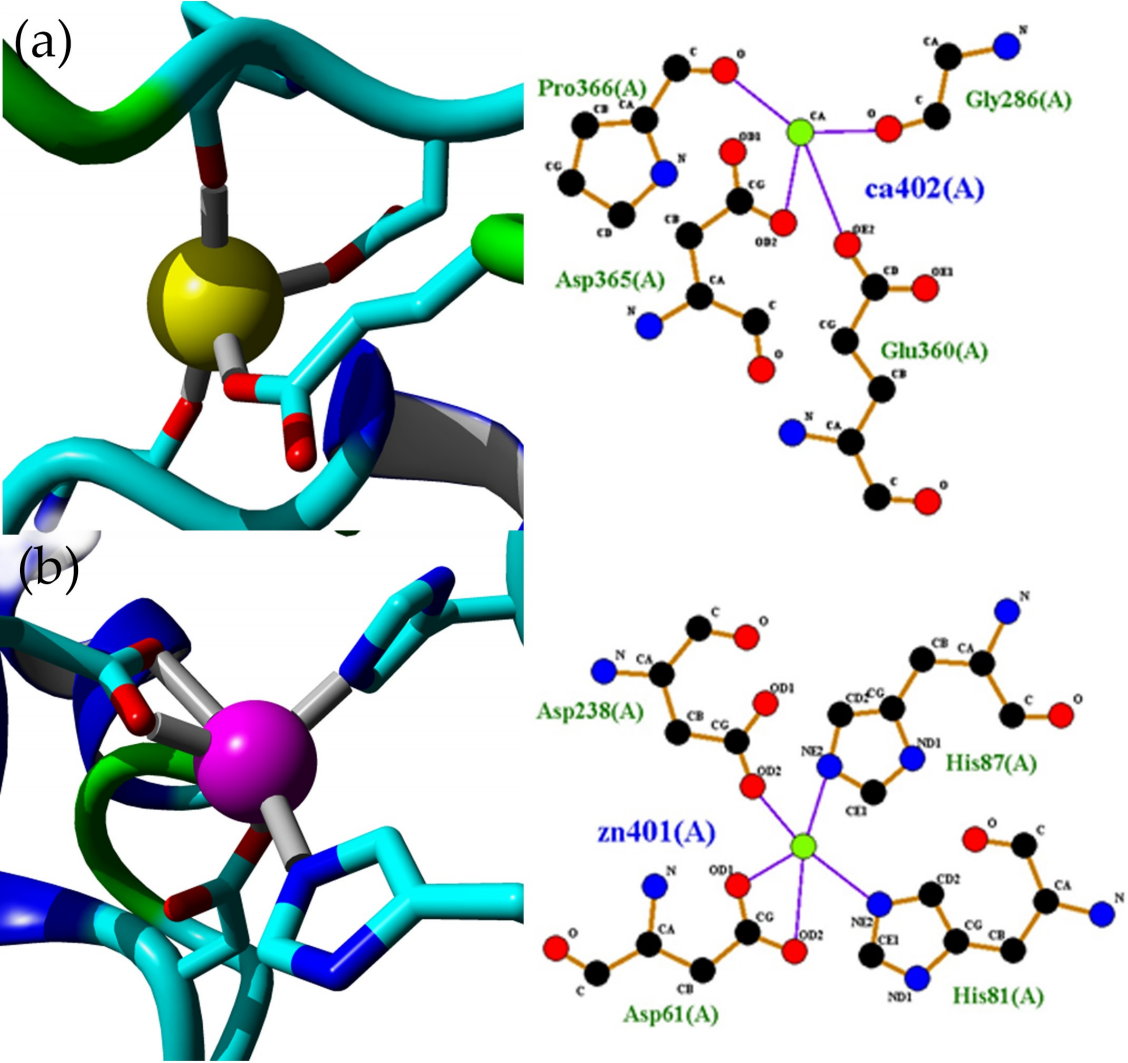
Metal-binding sites in 5M mutant crystal structure. (a) Calcium-binding site in chain A of 5M mutant crystal structure. The calcium-binding site composed of Gly286, Glu360, Asp365, and Pro366. A similar coordination was observed in chain B. (b) Zinc-binding sites in chain A of 5M mutant crystal structure. Zinc-binding site in both chains A and B composed of elements Asp61, Asp238, His87, and His81 in tetrahedral coordination. Hydrogen bonds color in the purple line. Metal ions color in green. The images were generated via YASARA software and LigPlot+ [27, 28].

The 5M mutant lipase has a total of 21 α-helices and 11 β-sheets in its 3D crystal structures. The active sites of 5M mutant lipase composed of Ser113, His358, and Aps317. A similar composition of the catalytic triad was found in the crystal structure of *Geobacillus thermoleovorans* lipase [36]. Both molecules in the asymmetric unit were highly similar, and the superimposition of alpha carbon atoms gave a root-mean-square deviation (RMSD) of 0.7789 Å (Fig 5). The increased value of RMSD was found to be located at the surface region, indicating the high flexibility of 5M mutant structure. The RMSD of the superposition of two different conformations from similar protein might be affected by the mobile region, containing flexible structures such as loops and hinged domains [37]. Additionally, the low resolution also contributes to the high values of RMSD of comparison structures. As noted by Carugo [38], two structures with high resolutions of 1.7 Å, had a small RMSD value, while the RMSD value hugely increased when low resolutions of the two structures were compared. Each asymmetric unit of the 5M mutant lipase crystal structure contained zinc- and calcium-binding sites (Fig 6). The zinc-binding sites in both chains composed of residues Asp61, Asp238, His87, and His81 in a tetrahedral formation. The calcium-binding site was coordinated by the residues Gly286, Glu360, Asp365, and Pro366. Other lipases such as thermostable lipase from *B*. *stearothermophilus* P1 have also reported zinc- and calcium-binding sites in their structures [34].

The biochemical and biophysical analysis indicated that the 5M mutant had a decline in thermostability and half-life after being treated at high temperature compared to its wild-type, T1 lipase [20]. For this particular reason, a comprehensive structural analysis of a 5M mutant lipase crystal structure was conducted to determine the differences in structural conformation that led to structural destabilization. The analysis of molecular interaction using YASARA software showed that the 5M mutant lipase consisted of a low number of molecular interactions (Table 2).

**Table 2:** Molecular interactions in 5M mutant crystal structure.

Interactions	T1 lipase		5M mutant	
	Chain A	Chain B	Chain A	Chain B
Hydrogen bonds	311	310	288	288
Ion interactions	22	25	20	17
Hydrophobic interactions	688	691	681	682
Aromatic interactions (π-π)	41	41	41	41
Cation- π interactions	11	9	12	10

The mutations of amino acids in the 5M mutant caused the destruction of the ion interactions and hydrogen bonds inside the lipase structure, as shown by decreasing ion interactions and hydrogen bonds number. The structural analysis of the 5M mutant crystal structure is in correlation with the experimental data reported previously. The study expected that amino acid substitution was capable of adding more interactions by increasing the interactions between the substituted amino acids. However, instead of enhancing the interactions, the amino acid substitution caused the destruction of its molecular interactions. Despite having additional hydrogen bonds due to mutations, the 5M mutant was found to lose several hydrogen bonds in its structure, as the number of hydrogen bonds analysis had an overall decrease. The mutant also experienced a decline in ion-pair interaction values, having only 20 pairs of ion interactions in chain A and 17 pairs of interactions in chain B. These results explained the loss in the thermostability of the 5M mutant, which also resulted in decreased melting temperature of this enzyme. A comparison of the mesophilic and hyper-thermophilic structures of glutamate dehydrogenase (GDH) revealed an increased number of ion-pair interactions and ion pair networks in the hyperthermophile structure from *Pyrococcus furiosus* and represent a major stabilizing feature of enzymes to extreme temperatures [39]. This observation suggests that the number of ion-pair interactions and hydrogen bonds are key features involved in the determination of protein thermostability. According to Zhao et al. [40], structural analysis and biochemical assays of lipase from marine *Streptomyces* sp. strain W007 (MAS1) revealed that salt bridges also affect thermostability. In conclusion, amino acid substitutions in the 5M mutant failed to increase the interactions of molecules within the protein when the overall structure turns to destruction. The introduction of more than one point mutations in the protein is rather complicated, as additivity and non-additivity arise due to interactions with one another and causes either cooperative (positive) or antagonistic (negative) effects. As an example, an attempt to increase the thermostability of α-amylase from *Bacillus licheniformis* (BLA) is successful in combining positive mutations [41]. Meanwhile, in another study, the negative effect was observed in the combination of point mutations of S185D/Q188T and G104D/A156R of *Aspergillus aculeatus* β-1,4-galactanase, which reduced stability [42].

### Substrate specificity and hydrolysis in various natural oils

A substrate specificity study was conducted using various carbon length of *p*-nitrophenyl includes *p*NP-acetate (C2), *p*NP-butyrate (C4), *p*NP-decanoate (C10), *p*NP-dodecanoate (C12), *p*NP-myristate (C14), and *p*NP-palmitate (C16). Based on the results obtained (Table 3), 5M mutant showed higher levels of activity towards long-chain carbon *p*NP-C10 to -C16 with preference towards *p*NP-dodecanoate (C10). The results indicate that 5M mutant lipase preferred long-chain carbon for hydrolysis, with almost zero activities detected at short-chain carbon *p*NP-C2 and -C4, indicating that the enzyme is a real lipase. Similar to our findings, lipase from *Bacillus thermoamylovorans* BHK67 showed desirable longer-chain substrate specificity with optimum activity in a substrate *p*NP-C16 [43]. On the contrary, lipase of *Streptomyces* sp. strain W007 (MAS1) has a low activity towards long-chain carbon and preferred middle-chain carbon *p*NP-C6 to -C10 [44].

**Table 3:** Hydrolysis of 5M mutant lipase.

Substrates	Activity (U/mL)
*p*NP-esters	
*p*NP-acetate (C2)	0 ± 9^a^
*p*NP-butyrate (C4)	3.7 ± 19.3^a^
*p*NP-decanoate (C10)	261.2 ± 9.3^c^
*p*NP-dodecanoate (C12)	236.8 ± 18.1^b,c^
*p*NP-myristate (C14)	193.3 ± 9.0^b^
*p*NP-palmitate (C16)	223.0 ± 18.2^b,c^
Natural substrates	
Olive oil (C18:1)	279.4 ± 0.7^c^
Coconut oil (C12:0)	171.8 ± 1.4^a^
Rice Bran oil (C18:1)	298.7 ± 2.8^c^
Soy oil (C18:1)	295.0 ± 1.0^c^
Corn oil (C18:2)	321.3 ± 2.2^d^
Sunflower oil (C18:2)	214.5 ± 0.3^b^
Canola oil (C18:1)	283.8 ± 0.9^c^

Mean values (x ± s.e.) followed by the same letters were not significantly different (Tukey test, p ≤ 0.05).

The effect of natural oils on lipase activity was studied by comparing the hydrolytic activity of natural oils to olive oil. The efficiency of the hydrolysis reaction of lipase varied with the olive oil examined. Generally, lipase demonstrated high activity in C18 oils. Among all the natural oils, 5M lipase showed preferences for rice bran oil, soy oil, and corn oil, with relative activity levels of 106.9%, 105.6%, and 115.0%, respectively. The lipase loses 40% of its ability to hydrolase C12 oil as shown in the decreasing hydrolysis rate in coconut oil, due to the free fatty acid content in coconut oil, which contains shorter fatty acid carbon chain length (C8). Meanwhile, lipase AMS3 from *Pseudomonas* sp. exhibited high hydrolytic activities towards natural oils of sunflower oil, coconut oil, and canola oil, but declining activity for hydrolase corn oil [45].

**Fig 7:**
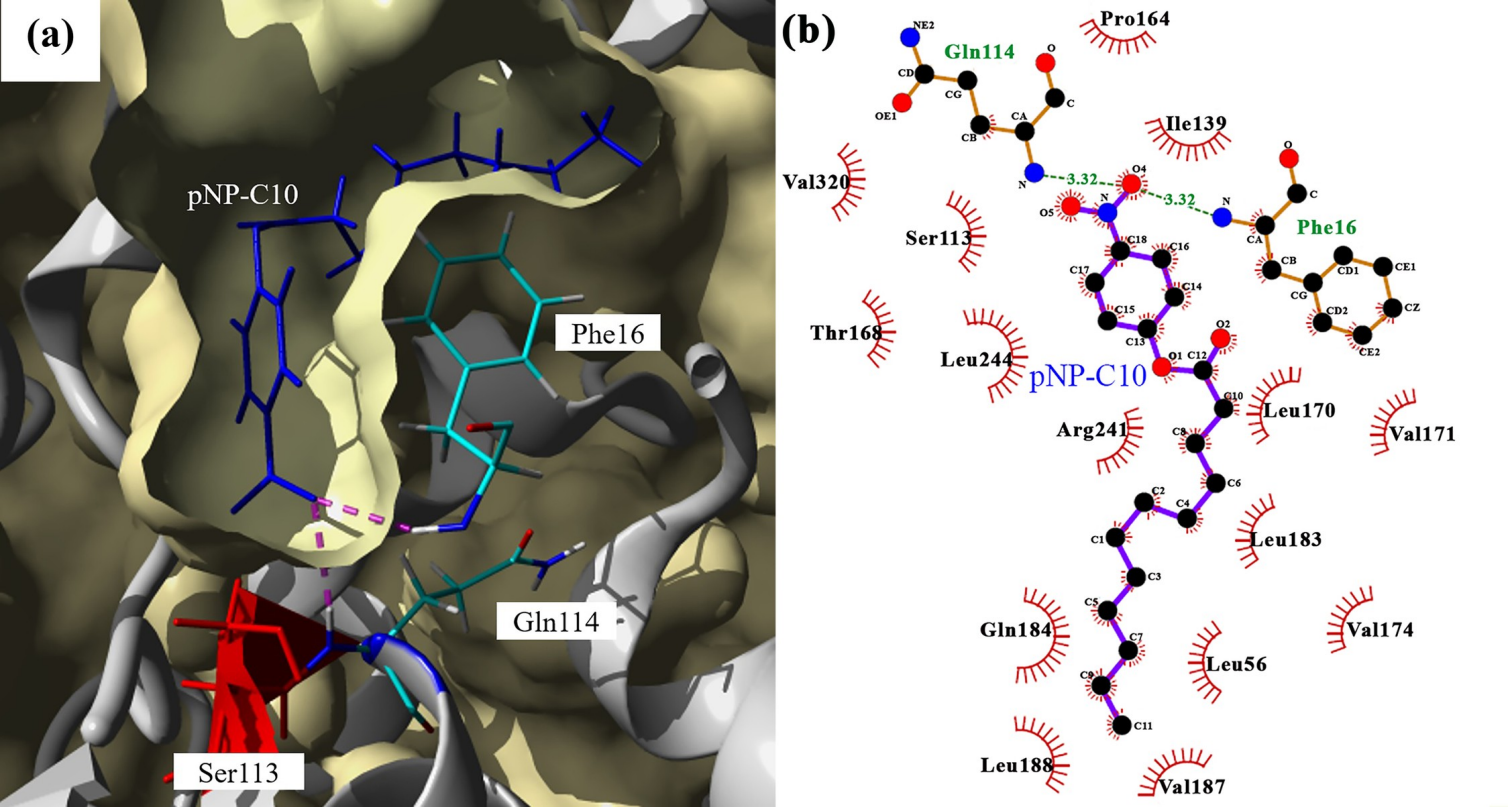
Molecular docking analysis of 5M lipase. (a) Molecular surface representation of 5M lipase with *p*NP-C10. Blue stick represents ligand. Oxyanion hole residues color in element. The red stick represents the catalytic residue, Ser113. Hydrogen bonds colored in magenta. (b) 2D interaction analysis of 5M lipase with *p*NP-C10. Hydrogen bonds are represented by a green dashed line, and hydrophobic interactions are represented by red spokes radiating towards the ligand. Ligands are represented in purple. C, N, and O atoms are represented in black, blue, and red, respectively. The images were generated via YASARA and LigPlot+ [27, 28].

The active site of 5M is located at the center of the structure covered by two alpha-helices functioning as the lid. The active site is composed of acid-base-nucleophile (Asp317-His358-Ser113) amino acids, which formed a deep tunnel, which allowed the long-chain substrate to enter it. The ligand-protein binding process began with the movement of the ligand through the tunnel to the active site followed by the binding of the ligand on the active site. Hence, the binding of the substrate depends on both binding tunnel and active site fitting. The docking analysis on various substrates with short chain (*p*NP-C2 and -C4) and long-chain (*p*NP-C10, -C12, -C14 and -C16) showed that all substrates migrated through the tunnel to the active site, however, the failure of 5M in catalyzing of short-chain substrate due to the fitting of the substrate on the active site during the catalysis process. An observation of the presence of various lengths of substrates showed a perfect fit for long-chain substrates, such as *p*NP-C10 to -C16. The docking analysis indicated that *p*NP-C2 and -C4 did not interact with any catalytic residues, answering why there is no activity for these substrates. The protein-ligand docking showed binding energy of 5.08 and 6.07 kcal/mol for C2 and C4, respectively, after clustering 25 runs. The distance between the catalytic Ser113 and the substrates *p*NP-C2 and -C4 were 4.27 Å and 3.22 Å. According to Chen et al. [46], the distance between the catalytic residues and substrate will influence the substrate binding. As reported by Mazlan et al. [31], high binding energies were also shown by *p*NP-C6 and -C8 substrates when docking analysis was performed on EstJ15 esterase, however, the substrates were not interacting with the catalytic Ser residue, explaining why EstJ15 esterase did not catalyze these substrates. Substrate C10 and C16 showed the highest binding energies compared to other substrates with 7.56 and 7.76 kcal/mol, respectively. The analysis showing that substrate of *p*NP-C10 formed hydrogen bonds with the oxyanion hole residues Gln114 and Phe16 (Fig 7A and 7B), whereas substrates *p*NP-C14 and -C16 created hydrogen bonds interaction with one oxyanion hole residue, Gln114, and Phe16, respectively, showing that 5M mutant has a high performance to catalyze long-chain residues. These interactions with surrounding residues near the catalytic site, benefiting the catalysis reaction. Interactions of the substrate with surrounding amino acids near the Serine of the catalytic site and carbonyl atom of the ester will be beneficial to the catalysis reaction [46].

### Molecular dynamics simulation

**Fig 8:**
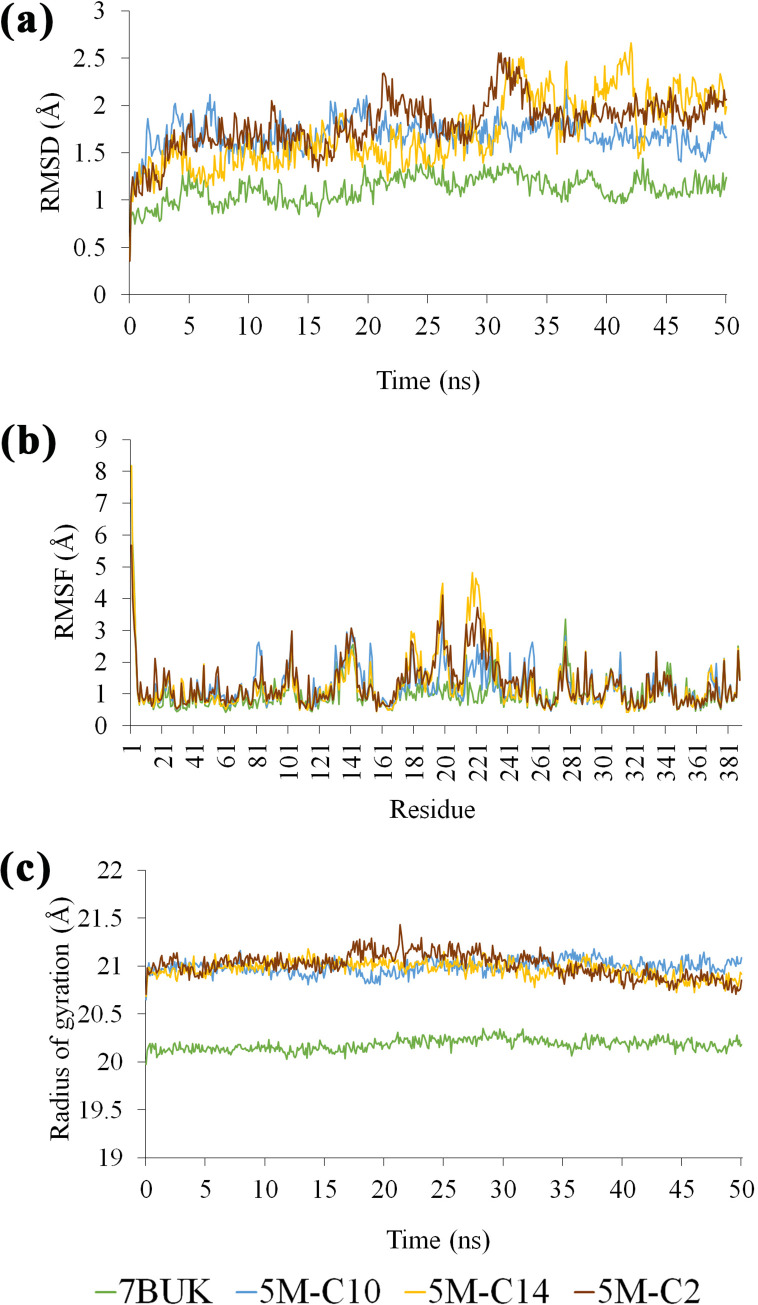
MD simulation results. (a) RMSDs of free 5M (7BUK) and 5M-substrates complex system during 50 ns MD simulation. (b) RMSF values of free 5M (7BUK) and 5M-substrates complex system were plotted against residue numbers. (c) Rg values free 5M (7BUK) and 5M-substrates complex system during 50 ns MD simulation.

**Fig 9:**
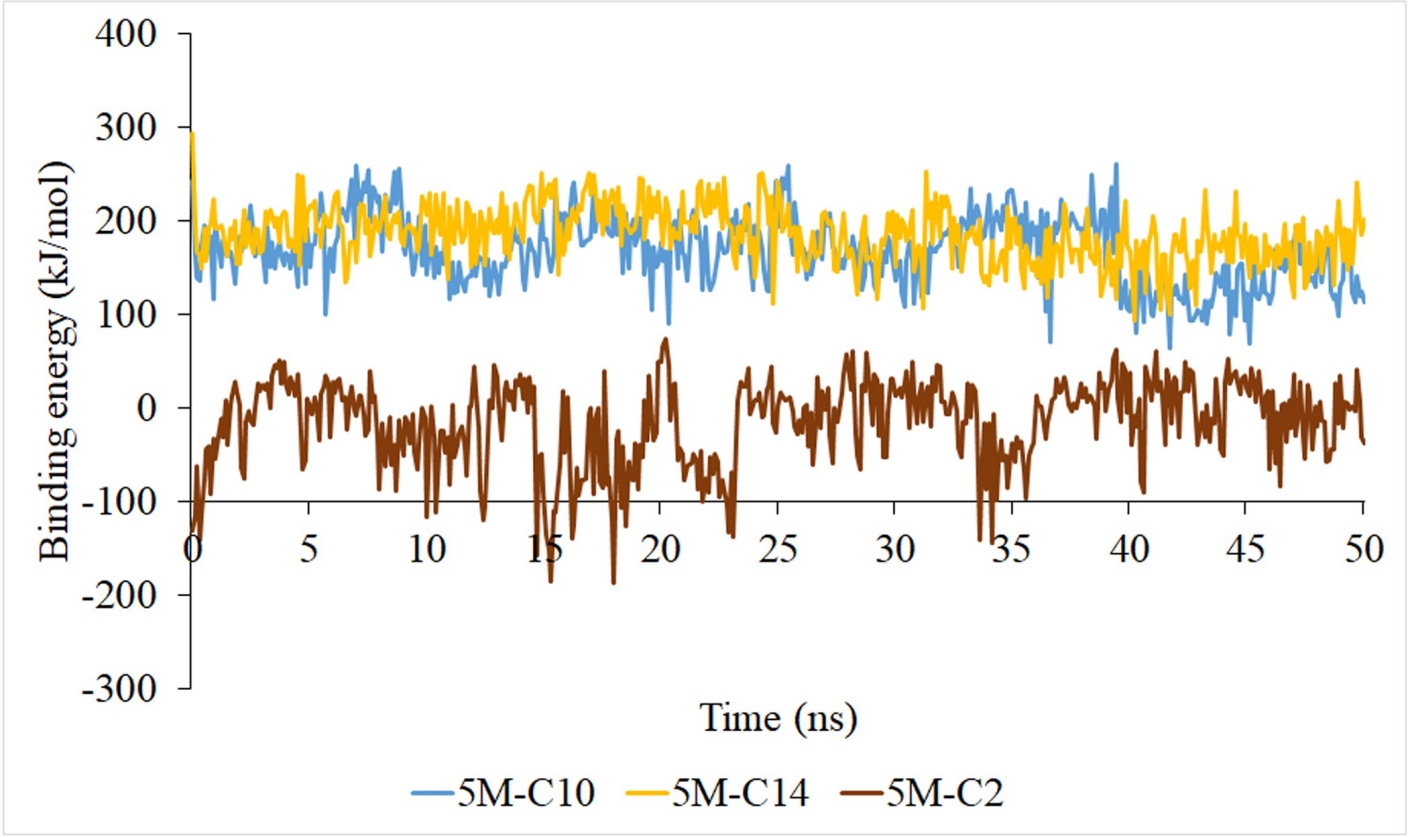
Binding energy of 5M-substrate complexes.

The starting structure of 5M-C2, 5M-C10, and 5M-C14 complex systems were obtained from molecular docking results and was then used in MD simulation. The root mean square deviation (RMSD), root mean square fluctuation (RMSF), radius of gyration (Rg), and binding energy were analyzed from trajectories to understand the dynamical properties and stability of the complex systems. RMSD values of the Cα atoms in the complexes were represented against simulation time from 0 to 50 ns (Fig 8A). RMSD values of 5M-C2 system were stable at the first 20 ns, then starting to fluctuate after 20 ns of simulation. A similar situation shows by system 5M-C14 in which stable during the first 30 ns of simulation and the values fluctuated after 30 ns. However, RMSD values of 5M-C10 system and free 5M lipase (7BUK) were stable throughout the simulation. The RMSD for 5M-substrate complexes is a little higher than the free 5M lipase, indicating that the motions of complexes increased due to the binding of substrate. A similar outcome was shown by the root mean square fluctuations (RMSF) data. RMSF values used to analyze the mobility and fluctuation of the residues, and the data can be used to study the dynamic movement of the protein-ligand system [47]. The RMSF values of residue during the 50 ns of simulation were plotted against residue numbers. As shown in Fig 8B, free 5M lipase (7BUK) was less flexible than the complexes of 5M-substrates (5M-C2, 5M-C10, and 5M-C14). The increasing flexibility in the complex structures happened at the amino acids located at the lid of the lipase. The radius of gyration (Rg) provides an overview of the dimension and compactness of a protein [48]. As shown in Fig 8C, Rg values of free 5M lipase (7BUK), which are in a close conformation were lower than 5M-substrate complexes and plateau throughout the simulation. The Rg value of the 5M-substrate complexes remained above the value of free 5M lipase, which signifies that the structure of complexes becomes loose due to substrate binding. Fig 9 shows the values of the binding energy of a ligand to the receptor corresponding to each time point in the molecular dynamic simulation process. As seen from the figure, the binding energy of 5M-C10 and 5M-C14 are higher than 5M-C2 indicates the long-chain ligands are tightly bound to the receptor (5M). The negative value of the binding energy in 5M-C2 system shows that less energy is required to separate the ligand C2 and 5M, which means the ligand is not strongly binds to the active site, thus explaining why 5M lipase did not catalyze the short chain substrates.

## Conclusions

The 3D crystal structure of 5M mutant lipase was successfully elucidated. The elucidation of the 5M mutant crystal structure revealed missing interactions in the structure and decrease in hydrogen bonds number contributed to the destabilization of the mutant structure compared to the wild type T1 lipase, thus reducing the stability of the 5M mutant lipase to withstand extreme conditions such as high temperature. From this study, it can be concluded that hydrogen bonds and ion interactions are crucial in maintaining the protein stability of T1 lipase. The 5M lipase has a strong capability to catalyze long-chain carbon and shows preference towards *p*NP-C10.

## Supporting information

S1 Raw images (PDF)

S1 File (PDF)

S2 File (PDB)
