# Supplementary material for: Structure elucidation and docking analysis of 5M mutant of T1 lipase Geobacillus zalihae
Source: PLoS One. 2021 Jun 1;16(6):e0251751. doi: 10.1371/journal.pone.0251751 (PMC8168862; doi:10.1371/journal.pone.0251751)
Supplement: S1 Raw images — (PDF) [file pone.0251751.s001.pdf]

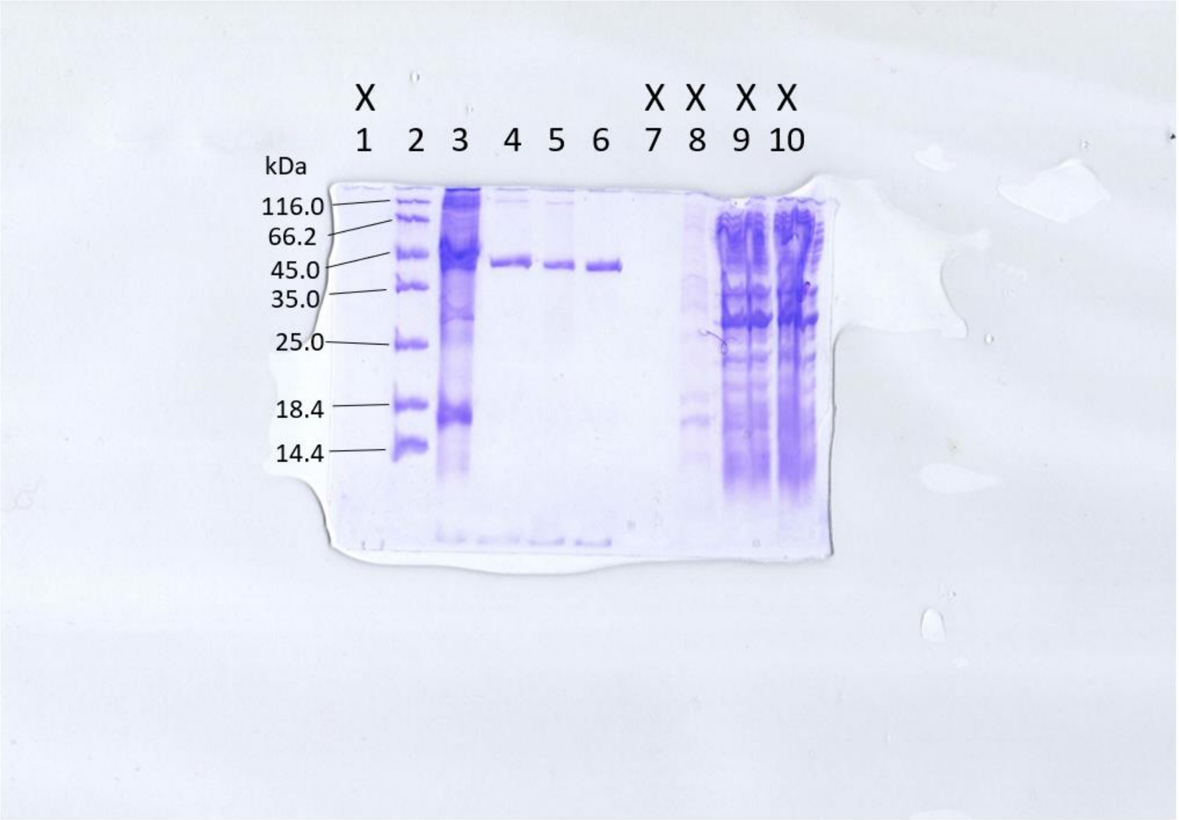

1. Labelling:
   1- Empty
   2-protein marker
   3-crude enzyme
   4- purified enzyme from affinity chromatography
   5-tag-cleaved enzyme after dialysis
   6-purified enzyme after ion exchange chromatography (second step)
   7-empty
   8-unidentified sample (other enzyme)
   9-unidentified sample (other enzyme)
   10-unidentified sample (other enzyme)

2. Samples with label X (lanes number 1, 7 – 10) are not included in the manuscript. Only samples 2-6 were used in this study.
3. This SDS gel (samples loaded on lane 2-6) was using to generate image for Figure 1, page: 6, Line: 229 (Fig 1. SDS-Page analysis of 5M mutant lipase.)
4. The image was captured using scanner of HP LaserJet Pro M1212nf Multifunction Printer.
