# Supplementary material for: Structure elucidation and docking analysis of 5M mutant of T1 lipase Geobacillus zalihae
Source: PLoS One. 2021 Jun 1;16(6):e0251751. doi: 10.1371/journal.pone.0251751 (PMC8168862; doi:10.1371/journal.pone.0251751)
Supplement: S1 File — (PDF) [file pone.0251751.s002.pdf]

Full wwPDB X-ray Structure Validation Report (i)

Apr 16, 2020 – 04:03 PM JST

PDB ID	:	7BUK
Title	:	T1 lipase mutant - 5M (D43E/T118N/E226D/E250L/N304E)
Deposited on	:	2020-04-07
Resolution	:	2.64 Å(reported)

This is a Full wwPDB X-ray Structure Validation Report.

This report is produced by the wwPDB biocuration pipeline after annotation of the structure.

We welcome your comments at *validation@mail.wwpdb.org*
A user guide is available at
https://www.wwpdb.org/validation/2017/XrayValidationReportHelp
with specific help available everywhere you see the (i) symbol.

The following versions of software and data (see references (i)) were used in the production of this report:

MolProbity	:	4.02b-467
Xtriage (Phenix)	:	1.13
EDS	:	2.10.1
Percentile statistics	:	20171227.v01 (using entries in the PDB archive December 27th 2017)
Refmac	:	5.8.0158
CCP4	:	7.0.044 (Gargrove)
Ideal geometry (proteins)	:	Engh & Huber (2001)
Ideal geometry (DNA, RNA)	:	Parkinson et al. (1996)
Validation Pipeline (wwPDB-VP)	:	2.10.1

CONFIDENTIAL VALIDATION REPORT

1 Overall quality at a glance (i)

The following experimental techniques were used to determine the structure:
X-RAY DIFFRACTION

The reported resolution of this entry is 2.64 Å.

Percentile scores (ranging between 0-100) for global validation metrics of the entry are shown in the following graphic. The table shows the number of entries on which the scores are based.

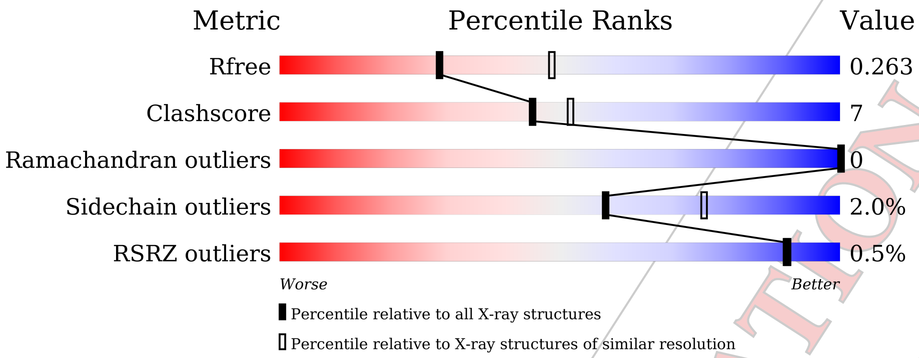

Metric	Whole archive (#Entries)	Similar resolution (#Entries, resolution range(Å))
R_{free}	111664	1191 (2.66-2.62)
Clashscore	122126	1233 (2.66-2.62)
Ramachandran outliers	120053	1216 (2.66-2.62)
Sidechain outliers	120020	1216 (2.66-2.62)
RSRZ outliers	108989	1175 (2.66-2.62)

The table below summarises the geometric issues observed across the polymeric chains and their fit to the electron density. The red, orange, yellow and green segments on the lower bar indicate the fraction of residues that contain outliers for >=3, 2, 1 and 0 types of geometric quality criteria respectively. A grey segment represents the fraction of residues that are not modelled. The numeric value for each fraction is indicated below the corresponding segment, with a dot representing fractions <=5% The upper red bar (where present) indicates the fraction of residues that have poor fit to the electron density. The numeric value is given above the bar.

Mol	Chain	Length	Quality of chain
1	A	406	% 80% 15% 5%
1	B	406	77% 17% • 5%

CONFIDENTIAL VALIDATION REPORT

2 Entry composition (i)

There are 4 unique types of molecules in this entry. The entry contains 6299 atoms, of which 0 are hydrogens and 0 are deuteriums.

In the tables below, the ZeroOcc column contains the number of atoms modelled with zero occupancy, the AltConf column contains the number of residues with at least one atom in alternate conformation and the Trace column contains the number of residues modelled with at most 2 atoms.

- Molecule 1 is a protein called Lipase.

Mol	Chain	Residues	Atoms					ZeroOcc	AltConf	Trace
1	A	387	Total 3056	C 1935	N 542	O 571	S 8	0	0	0
1	B	387	Total 3056	C 1935	N 542	O 571	S 8	0	0	0

- Molecule 2 is ZINC ION (three-letter code: ZN) (formula: Zn).

Mol	Chain	Residues	Atoms		ZeroOcc	AltConf
2	B	1	Total 1	Zn 1	0	0
2	A	1	Total 1	Zn 1	0	0

- Molecule 3 is CALCIUM ION (three-letter code: CA) (formula: Ca).

Mol	Chain	Residues	Atoms		ZeroOcc	AltConf
3	B	1	Total 1	Ca 1	0	0
3	A	1	Total 1	Ca 1	0	0

- Molecule 4 is water.

Mol	Chain	Residues	Atoms		ZeroOcc	AltConf
4	A	98	Total 98	O 98	0	0
4	B	85	Total 85	O 85	0	0

CONFIDENTIAL VALIDATION REPORT

3 Residue-property plots ⓘ

These plots are drawn for all protein, RNA and DNA chains in the entry. The first graphic for a chain summarises the proportions of the various outlier classes displayed in the second graphic. The second graphic shows the sequence view annotated by issues in geometry and electron density. Residues are color-coded according to the number of geometric quality criteria for which they contain at least one outlier: green = 0, yellow = 1, orange = 2 and red = 3 or more. A red dot above a residue indicates a poor fit to the electron density (RSRZ > 2). Stretches of 2 or more consecutive residues without any outlier are shown as a green connector. Residues present in the sample, but not in the model, are shown in grey.

• Molecule 1: Lipase

Chain A:

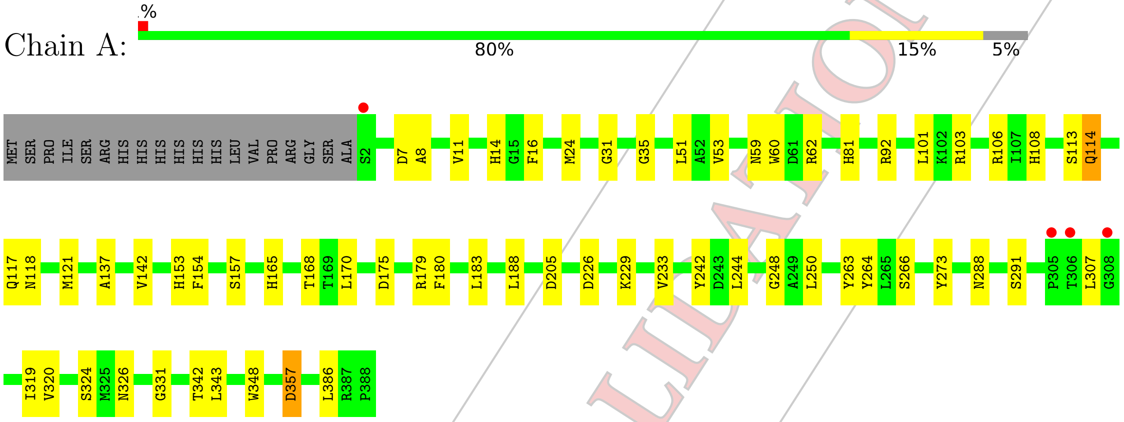

• Molecule 1: Lipase

Chain B:

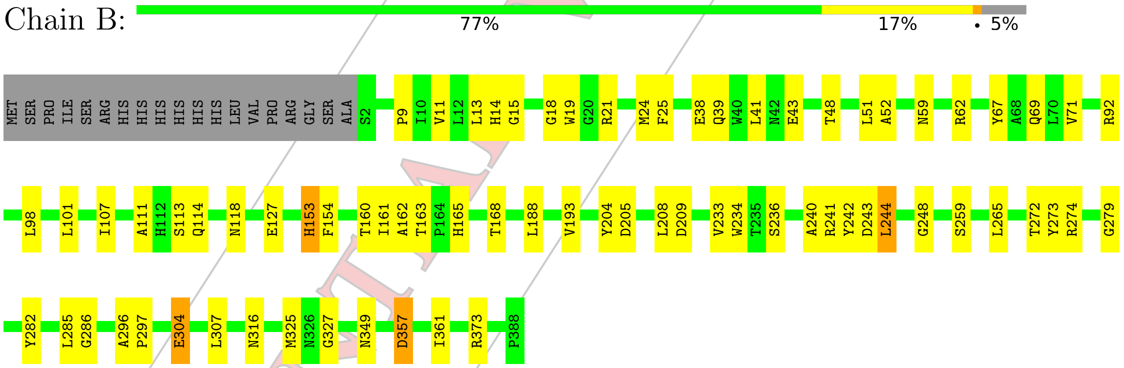

CONFIDENTIAL VALIDATION REPORT

4 Data and refinement statistics (i)

Property	Value	Source
Space group	C 1 2 1	Depositor
Cell constants<br>a, b, c, α, β, γ	117.59Å 81.27Å 99.52Å<br>90.00° 97.32° 90.00°	Depositor
Resolution (Å)	35.10 – 2.64<br>35.07 – 2.64	Depositor<br>EDS
% Data completeness<br>(in resolution range)	90.6 (35.10-2.64)<br>90.7 (35.07-2.64)	Depositor<br>EDS
R_{merge}	0.06	Depositor
R_{sym}	(Not available)	Depositor
$< I/\sigma(I) >$ [1]	5.97 (at 2.65Å)	Xtriage
Refinement program	REFMAC 5.8.0238	Depositor
R, R_{free}	0.184 , 0.262<br>0.189 , 0.263	Depositor<br>DCC
R_{free} test set	1189 reflections (4.79%)	wwPDB-VP
Wilson B-factor (Å^2)	17.0	Xtriage
Anisotropy	0.503	Xtriage
Bulk solvent k_{sol}(e/Å^3), B_{sol}(Å^2)	0.34 , 23.7	EDS
L-test for twinning[2]	$< \|L\| > = 0.49$, $< L^2 > = 0.33$	Xtriage
Estimated twinning fraction	No twinning to report.	Xtriage
F_o,F_c correlation	0.92	EDS
Total number of atoms	6299	wwPDB-VP
Average B, all atoms (Å^2)	17.0	wwPDB-VP

Xtriage's analysis on translational NCS is as follows: *The largest off-origin peak in the Patterson function is 4.76% of the height of the origin peak. No significant pseudotranslation is detected.*

[1] Intensities estimated from amplitudes.
[2] Theoretical values of $< |L| >$, $< L^2 >$ for acentric reflections are 0.5, 0.333 respectively for untwinned datasets, and 0.375, 0.2 for perfectly twinned datasets.

CONFIDENTIAL VALIDATION REPORT

5 Model quality (i)

5.1 Standard geometry (i)

Bond lengths and bond angles in the following residue types are not validated in this section: ZN, CA

The Z score for a bond length (or angle) is the number of standard deviations the observed value is removed from the expected value. A bond length (or angle) with $|Z| > 5$ is considered an outlier worth inspection. RMSZ is the root-mean-square of all Z scores of the bond lengths (or angles).

Mol	Chain	Bond lengths		Bond angles	
		RMSZ	#$\|Z\|$ >5	RMSZ	#$\|Z\|$ >5
1	A	0.74	0/3140	0.90	2/4268 (0.0%)
1	B	0.72	0/3140	0.88	1/4268 (0.0%)
All	All	0.73	0/6280	0.89	3/8536 (0.0%)

There are no bond length outliers.

All (3) bond angle outliers are listed below:

Mol	Chain	Res	Type	Atoms	Z	Observed(^{o})	Ideal(^{o})
1	A	103	ARG	NE-CZ-NH1	5.22	122.91	120.30
1	B	304	GLU	CB-CA-C	5.08	120.57	110.40
1	A	103	ARG	NE-CZ-NH2	-5.07	117.76	120.30

There are no chirality outliers.

There are no planarity outliers.

5.2 Too-close contacts (i)

In the following table, the Non-H and H(model) columns list the number of non-hydrogen atoms and hydrogen atoms in the chain respectively. The H(added) column lists the number of hydrogen atoms added and optimized by MolProbity. The Clashes column lists the number of clashes within the asymmetric unit, whereas Symm-Clashes lists symmetry related clashes.

Mol	Chain	Non-H	H(model)	H(added)	Clashes	Symm-Clashes
1	A	3056	0	2924	39	0
1	B	3056	0	2924	46	0
2	A	1	0	0	0	0
2	B	1	0	0	0	0
3	A	1	0	0	0	0
3	B	1	0	0	0	0

Continued on next page...

CONFIDENTIAL VALIDATION REPORT

Continued from previous page...

Mol	Chain	Non-H	H(model)	H(added)	Clashes	Symm-Clashes
4	A	98	0	0	2	0
4	B	85	0	0	1	0
All	All	6299	0	5848	84	0

The all-atom clashscore is defined as the number of clashes found per 1000 atoms (including hydrogen atoms). The all-atom clashscore for this structure is 7.

All (84) close contacts within the same asymmetric unit are listed below, sorted by their clash magnitude.

Atom-1	Atom-2	Interatomic distance (Å)	Clash overlap (Å)
1:A:60:TRP:HA	1:A:121:MET:HE2	1.48	0.96
1:A:114:GLN:OE1	1:A:244:LEU:HD13	1.72	0.90
1:A:92:ARG:NH2	1:A:205:ASP:OD2	2.05	0.89
1:B:62:ARG:HH21	1:B:118:ASN:HD22	1.27	0.82
1:B:92:ARG:NH2	1:B:205:ASP:OD2	2.19	0.75
1:B:373:ARG:HD3	4:B:559:HOH:O	1.86	0.74
1:A:14:HIS:HE1	1:A:53:VAL:H	1.36	0.73
1:B:52:ALA:H	1:B:69:GLN:HE22	1.38	0.71
1:B:234:TRP:CE2	1:B:241:ARG:HD2	2.26	0.71
1:A:168:THR:HG21	1:A:319:ILE:O	1.90	0.70
1:A:11:VAL:CG1	1:A:51:LEU:HG	2.23	0.68
1:B:98:LEU:O	1:B:101:LEU:HG	1.97	0.64
1:B:240:ALA:O	1:B:244:LEU:HB2	1.98	0.63
1:A:60:TRP:HA	1:A:121:MET:CE	2.29	0.61
1:A:106:ARG:HH21	1:A:153:HIS:HE1	1.49	0.61
1:B:304:GLU:HG3	1:B:307:LEU:HB2	1.83	0.59
1:B:208:LEU:HD21	1:B:233:VAL:HG21	1.85	0.58
1:A:62:ARG:HH21	1:A:118:ASN:HD22	1.52	0.58
1:A:168:THR:HG23	1:A:320:VAL:HA	1.85	0.58
1:A:114:GLN:NE2	1:A:117:GLN:OE1	2.38	0.56
1:B:304:GLU:HG2	1:B:307:LEU:HD22	1.87	0.56
1:A:307:LEU:HD13	4:A:596:HOH:O	2.05	0.56
1:B:296:ALA:HB3	1:B:297:PRO:HD3	1.88	0.56
1:B:160:THR:HB	1:B:163:THR:OG1	2.05	0.55
1:A:175:ASP:N	1:A:175:ASP:OD1	2.40	0.55
1:A:168:THR:HG22	1:A:170:LEU:H	1.71	0.54
1:A:288:ASN:ND2	1:A:291:SER:H	2.04	0.54
1:A:179:ARG:HD2	4:A:595:HOH:O	2.07	0.54
1:A:137:ALA:HA	1:A:142:VAL:HG12	1.91	0.53
1:B:357:ASP:OD1	1:B:357:ASP:C	2.46	0.53
1:A:59:ASN:HD21	1:A:114:GLN:HE21	1.55	0.53

Continued on next page...

CONFIDENTIAL VALIDATION REPORT

Continued from previous page...

Atom-1	Atom-2	Interatomic distance (Å)	Clash overlap (Å)
1:B:127:GLU:OE2	1:B:153:HIS:HD2	1.92	0.52
1:B:208:LEU:CD2	1:B:233:VAL:HG21	2.39	0.52
1:B:273:TYR:CZ	1:B:282:TYR:HB2	2.45	0.52
1:A:8:ALA:HB3	1:A:108:HIS:CE1	2.46	0.51
1:B:52:ALA:H	1:B:69:GLN:NE2	2.05	0.51
1:B:39:GLN:NE2	1:B:43:GLU:OE1	2.44	0.50
1:B:242:TYR:O	1:B:248:GLY:HA3	2.11	0.50
1:B:15:GLY:HA3	1:B:113:SER:HB3	1.94	0.50
1:A:168:THR:CG2	1:A:319:ILE:O	2.60	0.49
1:B:13:LEU:O	1:B:111:ALA:HA	2.13	0.49
1:A:62:ARG:HE	1:A:118:ASN:ND2	2.10	0.49
1:B:114:GLN:HE22	1:B:240:ALA:HB1	1.78	0.49
1:A:266:SER:OG	1:A:326:ASN:O	2.21	0.48
1:B:14:HIS:CG	1:B:18:GLY:HA3	2.49	0.48
1:B:114:GLN:NE2	1:B:240:ALA:HB1	2.29	0.48
1:B:274:ARG:HG2	1:B:279:GLY:HA2	1.97	0.47
1:B:162:ALA:HA	1:B:325:MET:SD	2.54	0.47
1:B:41:LEU:HD12	1:B:48:THR:HG21	1.96	0.47
1:B:25:PHE:CE2	1:B:193:VAL:HG22	2.50	0.47
1:A:31:GLY:HA3	1:A:35:GLY:O	2.14	0.47
1:A:242:TYR:O	1:A:248:GLY:HA3	2.14	0.46
1:B:92:ARG:NH1	1:B:209:ASP:OD1	2.49	0.46
1:B:234:TRP:CD2	1:B:241:ARG:HD2	2.50	0.46
1:A:233:VAL:O	1:A:233:VAL:HG22	2.15	0.45
1:B:272:THR:HG21	1:B:316:ASN:ND2	2.31	0.45
1:B:265:LEU:HA	1:B:349:ASN:O	2.17	0.45
1:A:101:LEU:HD13	1:A:154:PHE:CD1	2.52	0.45
1:A:60:TRP:CD1	1:A:81:HIS:CE1	3.05	0.45
1:A:264:TYR:HB3	1:A:348:TRP:CE3	2.52	0.44
1:B:19:TRP:CE2	1:B:24:MET:HG3	2.53	0.44
1:A:226:ASP:HA	1:A:229:LYS:HD2	2.00	0.44
1:A:24:MET:CE	1:A:188:LEU:HD23	2.47	0.44
1:B:67:TYR:O	1:B:71:VAL:HG22	2.18	0.44
1:A:14:HIS:CE1	1:A:53:VAL:H	2.26	0.43
1:B:21:ARG:NH1	1:B:38:GLU:HB3	2.33	0.43
1:A:157:SER:HA	1:A:263:TYR:O	2.18	0.43
1:A:16:PHE:CD1	1:A:16:PHE:C	2.91	0.43
1:B:59:ASN:ND2	1:B:243:ASP:HB3	2.34	0.43
1:B:9:PRO:HG2	1:B:107:ILE:HG22	2.00	0.43
1:B:234:TRP:CZ2	1:B:241:ARG:HD2	2.53	0.42
1:A:250:LEU:HD12	1:A:331:GLY:HA3	2.02	0.42

Continued on next page...

CONFIDENTIAL VALIDATION REPORT

Continued from previous page...

Atom-1	Atom-2	Interatomic distance (Å)	Clash overlap (Å)
1:A:180:PHE:O	1:A:183:LEU:HB3	2.20	0.42
1:B:282:TYR:CD1	1:B:282:TYR:N	2.88	0.41
1:B:165:HIS:CD2	1:B:327:GLY:HA2	2.55	0.41
1:B:272:THR:HG21	1:B:316:ASN:HD21	1.84	0.41
1:B:161:ILE:CG2	1:B:361:ILE:HG12	2.50	0.41
1:A:165:HIS:HB2	1:A:324:SER:O	2.21	0.41
1:B:11:VAL:CG1	1:B:51:LEU:HG	2.50	0.41
1:B:161:ILE:HG21	1:B:361:ILE:HG12	2.03	0.41
1:A:273:TYR:CE2	1:B:236:SER:HB3	2.56	0.41
1:A:343:LEU:HD13	1:A:386:LEU:HD21	2.02	0.40
1:B:285:LEU:HD12	1:B:286:GLY:N	2.36	0.40
1:A:357:ASP:C	1:A:357:ASP:OD1	2.60	0.40

There are no symmetry-related clashes.

5.3 Torsion angles (i)

5.3.1 Protein backbone (i)

In the following table, the Percentiles column shows the percent Ramachandran outliers of the chain as a percentile score with respect to all X-ray entries followed by that with respect to entries of similar resolution.

The Analysed column shows the number of residues for which the backbone conformation was analysed, and the total number of residues.

Mol	Chain	Analysed	Favoured	Allowed	Outliers	Percentiles	
1	A	385/406 (95%)	364 (94%)	21 (6%)	0	100	100
1	B	385/406 (95%)	370 (96%)	15 (4%)	0	100	100
All	All	770/812 (95%)	734 (95%)	36 (5%)	0	100	100

There are no Ramachandran outliers to report.

5.3.2 Protein sidechains (i)

In the following table, the Percentiles column shows the percent sidechain outliers of the chain as a percentile score with respect to all X-ray entries followed by that with respect to entries of similar resolution.

The Analysed column shows the number of residues for which the sidechain conformation was

CONFIDENTIAL VALIDATION REPORT

analysed, and the total number of residues.

Mol	Chain	Analysed	Rotameric	Outliers	Percentiles	
1	A	318/335 (95%)	313 (98%)	5 (2%)	65	80
1	B	318/335 (95%)	310 (98%)	8 (2%)	50	70
All	All	636/670 (95%)	623 (98%)	13 (2%)	58	76

All (13) residues with a non-rotameric sidechain are listed below:

Mol	Chain	Res	Type
1	A	7	ASP
1	A	113	SER
1	A	114	GLN
1	A	342	THR
1	A	357	ASP
1	B	153	HIS
1	B	154	PHE
1	B	168	THR
1	B	188	LEU
1	B	204	TYR
1	B	244	LEU
1	B	259	SER
1	B	357	ASP

Some sidechains can be flipped to improve hydrogen bonding and reduce clashes. All (16) such sidechains are listed below:

Mol	Chain	Res	Type
1	A	14	HIS
1	A	39	GLN
1	A	59	ASN
1	A	108	HIS
1	A	118	ASN
1	A	153	HIS
1	A	172	ASN
1	A	254	GLN
1	A	288	ASN
1	B	69	GLN
1	B	114	GLN
1	B	118	ASN
1	B	153	HIS
1	B	172	ASN
1	B	216	GLN
1	B	254	GLN

CONFIDENTIAL VALIDATION REPORT

5.3.3 RNA (i)

There are no RNA molecules in this entry.

5.4 Non-standard residues in protein, DNA, RNA chains (i)

There are no non-standard protein/DNA/RNA residues in this entry.

5.5 Carbohydrates (i)

There are no carbohydrates in this entry.

5.6 Ligand geometry (i)

Of 4 ligands modelled in this entry, 4 are monoatomic - leaving 0 for Mogul analysis.

There are no bond length outliers.

There are no bond angle outliers.

There are no chirality outliers.

There are no torsion outliers.

There are no ring outliers.

No monomer is involved in short contacts.

5.7 Other polymers (i)

There are no such residues in this entry.

5.8 Polymer linkage issues (i)

There are no chain breaks in this entry.

CONFIDENTIAL VALIDATION REPORT

6 Fit of model and data (i)

6.1 Protein, DNA and RNA chains (i)

In the following table, the column labelled '#RSRZ> 2' contains the number (and percentage) of RSRZ outliers, followed by percent RSRZ outliers for the chain as percentile scores relative to all X-ray entries and entries of similar resolution. The OWAB column contains the minimum, median, 95th percentile and maximum values of the occupancy-weighted average B-factor per residue. The column labelled 'Q< 0.9' lists the number of (and percentage) of residues with an average occupancy less than 0.9.

Mol	Chain	Analysed	<RSRZ>	#RSRZ>2			OWAB(Å^2)	Q<0.9
1	A	387/406 (95%)	-0.47	4 (1%)	82	81	7, 13, 33, 64	0
1	B	387/406 (95%)	-0.40	0	100	100	8, 17, 31, 54	0
All	All	774/812 (95%)	-0.43	4 (0%)	90	90	7, 15, 32, 64	0

All (4) RSRZ outliers are listed below:

Mol	Chain	Res	Type	RSRZ
1	A	2	SER	4.5
1	A	305	PRO	3.0
1	A	306	THR	2.9
1	A	308	GLY	2.6

6.2 Non-standard residues in protein, DNA, RNA chains (i)

There are no non-standard protein/DNA/RNA residues in this entry.

6.3 Carbohydrates (i)

There are no carbohydrates in this entry.

6.4 Ligands (i)

In the following table, the Atoms column lists the number of modelled atoms in the group and the number defined in the chemical component dictionary. The B-factors column lists the minimum, median, 95th percentile and maximum values of B factors of atoms in the group. The column labelled 'Q< 0.9' lists the number of atoms with occupancy less than 0.9.

Mol	Type	Chain	Res	Atoms	RSCC	RSR	B-factors(Å^2)	Q<0.9
3	CA	B	402	1/1	0.96	0.09	36,36,36,36	0

Continued on next page...

CONFIDENTIAL VALIDATION REPORT

Continued from previous page...

Mol	Type	Chain	Res	Atoms	RSCC	RSR	B-factors(Å^2)	Q<0.9
3	CA	A	402	1/1	0.97	0.06	32,32,32,32	0
2	ZN	A	401	1/1	0.99	0.04	26,26,26,26	0
2	ZN	B	401	1/1	1.00	0.03	34,34,34,34	0

6.5 Other polymers (i)

There are no such residues in this entry.

CONFIDENTIAL VALIDATION REPORT
